# Prediction accuracy of femoral and tibial stress and strain using statistical shape and density model-based finite element models in paediatrics

**DOI:** 10.1007/s10237-025-02016-8

**Published:** 2025-10-13

**Authors:** Yidan Xu, Laura Carman, Thor F. Besier, Julie Choisne

**Affiliations:** 1 Auckland Bioengineering Institute, The University of Auckland, 70 Symonds Street, Auckland, New Zealand; 2 Department of Engineering Science and Biomedical Engineering, The University of Auckland, Auckland, New Zealand

**Keywords:** Finite element analysis, Statistical shape and density model, Femur, Tibia, Paediatric bone biomechanics

## Abstract

Computed tomography (CT)-based finite element (FE) models can non-invasively assess bone mechanical properties, but their clinical application in paediatrics is limited due to fewer datasets and models. Statistical Shape-Density Model (SSDM)-based FE models using statistically inferred shape and density have application to predict bone stress and strains; however, their accuracy in children remains unexplored. This study assessed the accuracy of stress–strain distributions estimated from SSDM-based FE models of paediatric femora and tibiae. CT-based FE models used geometry and densities derived from 330 CT scans from children aged 4–18 years. Paediatric SSDMs of the femur and tibia were used to predict bone geometries and densities from participants’ demographics and linear bone measurements. Forces during single-leg standing were estimated and applied to each bone. Stress and strain distributions were compared between the SSDM-based FE models and CT-based FE models, which served as the gold standard. The average normalized root-mean-square error (NRMSE) for Von Mises stress was 6% for the femur and 8% for the tibia across all cases. Principal strains NRMSE ranged from 1.2% to 5.5%. High correlations between the SSDM-based and CT-based FE models were observed, with determination coefficients ranging from 0.80 to 0.96. These results illustrate the potential of SSDM-based FE models for paediatric application, such as personalized implant design and surgical planning.

**Supplementary Information:**

The online version contains supplementary material available at 10.1007/s10237-025-02016-8.

## Introduction

Finite Element Analysis (FEA) is a structural mechanics approach that considers the geometry, material properties and forces to non-invasively understand the mechanical behaviour of bone. Finite Element (FE) models have been used to examine fracture risk (Qasim et al. 2016), fracture healing (Shefelbine et al. 2005), bone development (Koller et al. 2025) and bone remodelling (Meslier and Shefelbine 2023). However, the majority of these models investigate adult bone, so might not be directly applicable to paediatrics.

Paediatric orthopaedic conditions such as cerebral palsy, hip dysplasia, slipped capital epiphysis and idiopathic torsional deformities require age-specific knowledge of musculoskeletal structure and function. Previous finite element studies in children have demonstrated the potential value of FEA for investigating biomechanical outcomes (e.g. stress and strain distribution) of deformed bone and joints in children with conditions (KIM et al. 2005; Incze-Bartha et al. 2024; Bavil et al. 2025), demonstrating the potential clinical value of FEA. However, these studies typically relied on generic bone geometries and material properties derived from adult datasets (KIM et al. 2005; Carriero et al. 2011; Kainz et al. 2020; Incze-Bartha et al. 2023; Bavil et al. 2025). Generic FE models are less accurate in predicting stress and strain than subject-specific models in both adults and children (Taddei et al. 2006; Li et al. 2015; Martelli et al. 2015). Evaluating the biomechanical properties of bones using subject-specific FE models based on computed tomography (CT) data has shown high accuracy in predicting strains when compared to experimental data from biomechanical studies (Taddei et al. 2006; Gray et al. 2008; Trabelsi et al. 2011). Despite this accuracy, creating subject-specific FE models in clinical settings is limited by the time and cost associated with model generation, simulation and interpretation. Moreover, the radiation dose associated with CT imaging presents a significant concern for the paediatric population, limiting the CT-based FE models for clinical use in children (Brody et al. 2007; Granata et al. 2024). To overcome this limitation, statistical shape-density models (herein referred to as *SSDM*) have shown promising results in predicting specific bone geometry and bone mineral densities for FE models in both adults (Nicolella and Bredbenner 2012; Grassi et al. 2014, 2017; Nolte and Bull 2019; Steiner et al. 2021) and children (Xu et al. 2025) using sparse input data.

SSDMs can predict bone geometry and bone material properties for various bones/joints. The previous studies based on an adult population reported shape prediction root-mean-square errors (RMSE) ranging from 1.22 to 2.20 mm in the femur (Grassi et al. 2014), tibia (Bruce and Edwards 2023) and scapula (Soltanmohammadi et al. 2020). For these same studies, predicted bone mineral density RMSEs typically ranged from 0.050 g/cm^3^ to 0.117 g/cm^3^. Our previous study evaluated the prediction accuracy of a paediatric femoral SSDM and found an average shape prediction RMSE of 1.77 mm and density prediction RMSE of 0.101 g/cm^3^ (Xu et al. 2025). While these studies quantified geometry and bone mineral density prediction errors, it is unknown how these errors influence the stress–strain distributions estimated from FEA.

Several studies have attempted to generate FE models using predicted shape and density based on SSDM (herein referred to as SSDM-based FE models) to bridge this gap using adult bone data. A previous study generated SSDM-based FE models and validated their strain accuracy with in vitro experiments of eight femurs under six quasi-axial loading conditions (Grassi et al. 2014). Similarly, another study generated SSDM-based FE models based on a shape and density model of the femur and 2D clinical images to estimate strains under single-leg stance, comparing them to experiments of three femurs (Grassi et al. 2017). Their accuracy was evaluated by comparing estimated strains with experimental measurements, showing strong correlations (*R*^2^ ≥ 0.80). Despite the promising results, these experimental studies on adults were limited by small sample sizes (*n* = 3–8), which poses a challenge in accounting for the wide variability in geometry and density across individuals. In contrast, another study without experimental validation performed a direct, one-to-one comparison of stress and strain distributions between SSDM-based FE models and corresponding CT-based FE models, providing a more generalizable comparison with a larger cohort (Nolte and Bull 2019). Building on this approach, we propose to verify our model in a paediatric population, where variability in geometry and density is even greater due to growth.

SSDM-based FE models have been applied to predict hip fractures (Bryan et al. 2009; Bredbenner et al. 2014), bone strength (Grassi et al. 2017; Steiner et al. 2021) and strain (Bruce et al. 2022; Bruce and Edwards 2023). They have also been used to improve treatment and implant design (Day et al. 2022; Soltanmohammadi et al. 2022) in adult populations. However, SSDM-based FE models have not been applied in paediatric cohorts, where alternatives to CT-based modelling are critically needed due to radiation concerns. We developed a framework to generate personalized femoral and tibial FE models from SSDMs of 330 children aged 4–18 years, using an imaging-free approach. The aim of the current study was to evaluate whether these SSDM-based FE models can accurately reproduce stress and strain distributions compared to those obtained from CT-based FE models. This study had two main objectives; (1) to create a SSDM of the tibia as previously published for the femur (Xu et al. 2025) and (2) to evaluate the accuracy of SSDM-based FEA of the femur (Xu et al. 2025) and tibia (from objective 1), compared to CT-based FEA (gold standard).

## Methodology

### Cohort information

Post-mortem Computed Tomography (CT) scans of 330 children (136 F, age = 12 ± 5 years old [4–18], height = 148 ± 24 cm [96–192] and weight = 49 ± 22 kg [14–140]) were acquired from the Victorian Institute of Forensic Medicine (Melbourne, Australia) with ethics approval from the VIFM Ethics Committee (2023-Choisne-1143-2385/3) and the Auckland Health Research Ethics Committee at the University of Auckland (AH24671). Prior to autopsy, the VIFM obtained written consent from the individual’s legal guardian. All methods were performed in accordance with the relevant guidelines and regulations. A calibration phantom was included in the CT scans (Model 3 CT Calibration Phantom, Mindways Inc.) to facilitate the mapping of Hounsfield Units to bone mineral density. The slice thickness of the CT scans varied from 0.5 to 2 mm and the pixel spacing from 0.57 × 0.57 to 1.27 × 1.27 mm.

### Statistical shape and density model (SSDM)

The creation of the femoral SSDM is described in a previous study (Xu et al. 2025). A tibial SSDM was subsequently created using the same method. Briefly, 657 femora and 652 tibiae were semi-automatically segmented from the CT scans using Deep Segmentation (Formus Labs) and Mimics (Materialise v.23) (Carman et al. 2022). For both the femur and tibia, a surface template mesh (F, 5Y, 111 cm) was fitted to the rest of the dataset using radial basis functions to achieve nodal correspondence (Zhang et al. 2016; Carman et al. 2022). Based on these surface template meshes, 4-node tetrahedral template meshes were generated for the femur and tibia using TetGen (Si 2015), with an element edge length of 2 mm.

A convergence analysis was conducted using single-leg standing loads (Bergmann et al. 2010; Kutzner et al. 2010), applying boundary conditions identical to those described in Sect. 2.3.2 to the femur and tibia template meshes as well as their respective largest meshes (M, 17Y, 192 cm). Convergence was defined as the condition where the average Von Mises stress change was less than 1% upon further mesh refinement. As a result, the femur mesh consisted of 21,900 nodes and 122,964 elements, and the tibia mesh comprised 25,874 nodes and 150,164 elements. Mesh quality was assessed and reported in our previous paper (Xu et al. 2025) for the femur and in Supplementary Information S1 for the tibia. Furthermore, template volumetric meshes for the femur and tibia were independently morphed to the rest of the dataset, using ‘host-mesh’ fitting to achieve correspondence of the elements (Fernandez et al. 2004).

**Fig. 1:**
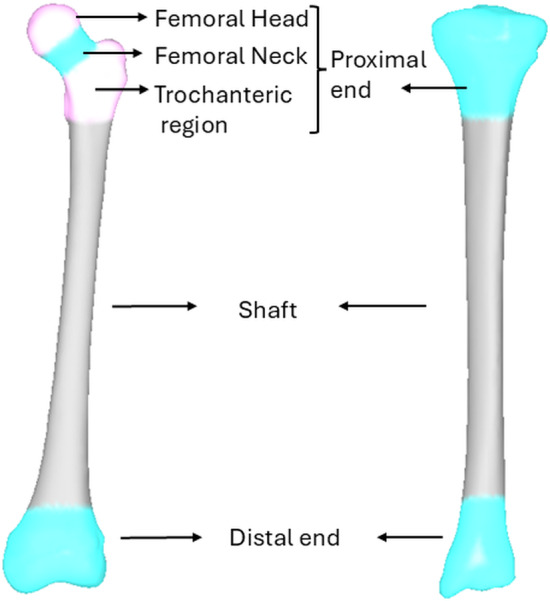
Regions of interests in the femur (left) and tibia (right)

Each volumetric mesh was imported into Bonemat (Rolling Version, Instituto Ortopedico Rizzoli, Bologna, Italy) (Taddei et al. 2007), to extract Hounsfield Units (HU) and convert these values to CT density using the relationship from the calibration phantom present in the CT scan. Element-wise apparent densities were then interpolated and assigned to each node by averaging the densities of the surrounding elements connected to that node. To standardize anatomical orientation for subsequent analysis, the femur and tibia bone segments were aligned with an International Society of Biomechanics coordinate system convention (Wu et al. 2002). For each model, the nodal coordinates (*X*, *Y* and *Z*) and density at each node were constructed as a point distribution matrix, where each individual’s coordinates and density variables were represented as one column vector *V* = (*X*_1_, *Y*_1_, *Z*_1_, *D*_1_, …, *X*_*n*_, *Y*_*n*_, *Z*_*n*_, *D*_*n*_)^T^, where n denotes the total number of nodes. This matrix was centred and then scaled by its standard deviation, ensuring that both shape and density contributed equally to the analysis. Principal component analysis (PCA) was then performed separately on the femoral and tibial standardized matrix to obtain mean models and PC weights (Jolliffe 2014; Zhang et al. 2016). These PC weights were subsequently used to train Partial Least Square Regression models against predictive variables, including demographic data (age, height, mass and sex) and linear bone measurements. For the femur, these measurements (Supplementary Information S2) included epicondylar width and femur length, and for the tibia, they included condylar width, malleolar width and tibial length (Carman et al. 2023). To evaluate the accuracy of each model, a leave-one-out (LOO) analysis was performed for the femur and tibia independently, predicting bone geometry and bone mineral density by excluding one participant at a time from the PCA training set to prevent bias. Predictive accuracy was estimated using root-mean-square error (RMSE) for each case between segmented surface and predicted nodes for shape, and between predicted nodal densities and CT-based densities. Density NRMSE for each model was calculated by normalizing the RMSE with its mean density value. To evaluate the relationship between prediction accuracy and age, errors in anatomical angle measurements—including femoral neck-shaft angle (NSA), anteversion angle (AA) and tibial torsion—were computed for each subject and analysed using Pearson correlation coefficients (see Supplementary Information S2, Fig. S3 for angular measurement definitions). For density, absolute errors between SSDM- and CT-based values were calculated for each anatomical region (proximal, shaft and distal) (Fig. 1), and Pearson correlation analysis was performed to assess age-related trends in prediction accuracy.

### Finite element model generation

#### Geometries and material properties

**Fig. 2:**
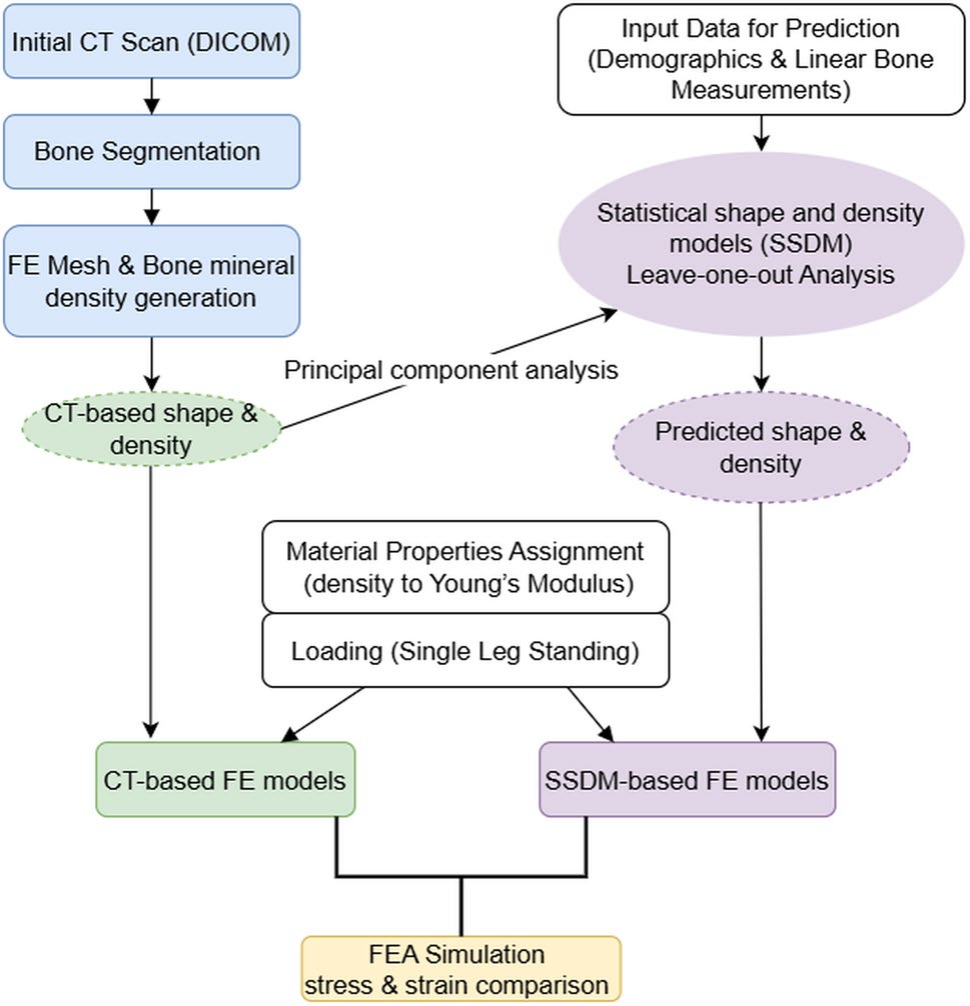
The flowchart representing the generation of the finite element models for the CT-based model and the SSDM-based model

For each individual in the dataset, two FE models were developed for the femur and tibia, a gold standard model using CT scan information and the second SSDM-based model using the statistical shape and density models predictions. The complete workflow for the generation of these FE models is illustrated in Fig. 2. The CT-based FE model was constructed using the segmented bone geometry from the CT scan and bone mineral densities directly derived from the CT scans (Sect. 2.2). The SSDM-based FE model was developed using bone geometry and nodal densities distribution predicted from the LOO analysis (Sect. 2.2).

The bone mineral density of each element ($\rho_{CT}$) in the SSDM-based FE model was assigned by averaging the nodal densities of its surrounding nodes. For both models, the elemental densities were transformed to Young’s modulus using the following equations.

##### Femur

The apparent density of bone ($\rho_{app}$) was estimated by assuming the following relationships with ash density ($\rho_{ash}$) and bone mineral density ($\rho_{CT}$), reported by Schileo et al. (2008):

1 $$\rho_{ash} = 0.8772\rho_{CT} + 0.07895$$

2 $$\rho_{app} = \frac{1}{0.6}\rho_{ash}$$

The Young’s modulus ($E$) for each element was then estimated based on the following relationship proposed by Morgan et al. (2003):

3 $$E_{Femur} = 6850\rho_{app}^{1.49} = 14664\left( {0.8772\rho_{CT} + 0.07895} \right)^{1.49}$$

##### Tibia

The Young’s modulus ($E$) in tibia for each element was estimated based on the following relationship proposed by Keyak et al. (1994):

4 $$\rho_{ash} = 1.06\rho_{CT} + 0.0389$$

5 $$E_{Tibia} = 11300\rho_{ash}^{1.9} = 11300\left( {1.06\rho_{CT} + 0.0389} \right)^{1.9}$$

#### Boundary conditions

For both models (CT- and SSDM-based), the boundary conditions were applied as follows. All tibiae and femora were previously aligned to the International Society of Biomechanics coordinate system recommendation for the femur and tibia (Wu et al. 2002).

##### Femur

**Fig. 3:**
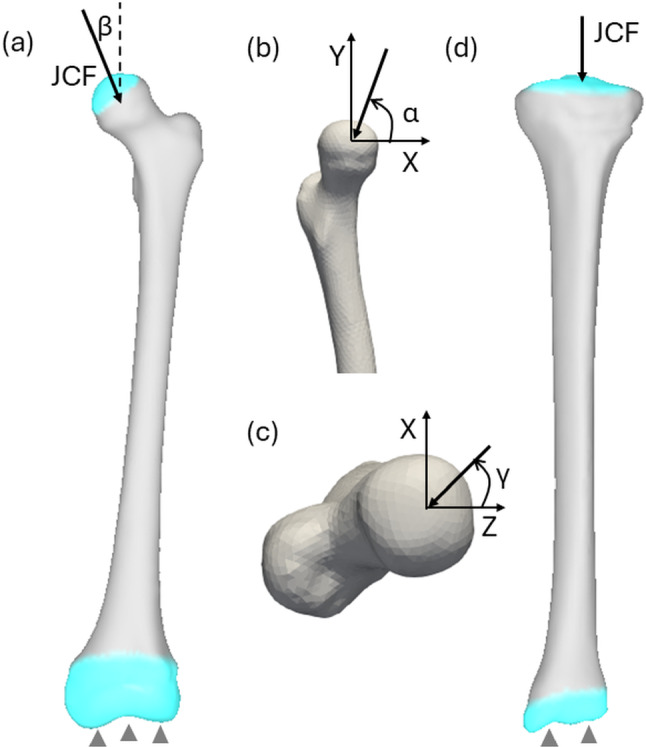
Boundary conditions applied to the femur **a** distal end was fully constrained, and hip joint contact force (JCF) was applied on the hemispheric area of femoral head; hip joint force angles are displayed in the frontal plane (**a**), sagittal plane (**b**) and transverse plane (**c**). Knee JCF and constraints were applied on the tibia (**d**)

The hip joint contact force with a magnitude of 2.4 times body weight was applied on the femoral head. This force represents the average peak contact force in the hip during single-leg standing from instrumented prostheses (Bergmann et al. 2010). This force was applied at angles of 84.3° (*α*) relative to the anterior axis in the frontal plane, 1.7° (*β*) relative to the superior axis in the sagittal plane and 30° (*γ*) relative to the condyle axis in the transverse plane, respectively (Fig. 3). The force was distributed across the outer hemispherical surface of the femoral head (Hölzer et al. 2013). The nodes at the distal end (lower 20% of the total length) of the femur were constrained in all degrees of freedom, while the nodes on the hip joint contact surface were restricted to move along the superior–inferior axis.

##### Tibia

A knee joint contact force of 2.5 times body weight was applied to the tibial plateaus in the inferior direction (Fig. 3d). This force represented the average peak contact force in the knee joint during single-leg standing, as reported by Kutzner et al. (2010) using instrumented prostheses. The nodes on the tibial plateaus were only allowed to move along the superior–inferior axis, while the distal end of the tibia was fixed in all degrees of freedom.

All simulations were performed in FEBio 4.3.0 with a quasi-static analysis (Maas et al. 2012).

### Comparison of stress–strain predictions

Mean values of both average and maximum Von Mises stress and first principal strain were computed across all models for proximal end, shaft and distal end. Correspondingly, mean relative errors were calculated by averaging the relative errors across all models. For each model, relative error was defined as:

$${\text{Relative}}\;{\text{ error}} \left( \% \right) = \frac{{\left( {{\text{SSDM value}} - {\text{CT value}}} \right)}}{{\text{CT value}}} \times 100\%$$

CT-based FEA and corresponding SSDM-based FEA outputs were compared to evaluate the ability of the predicted model to reproduce gold standard CT-based FEA stress and strain values. Von Mises stress differences were assessed at the element level using root-mean-square error (RMSE) and normalized root-mean-square error (NRMSE), which was normalized by the maximum stress within the regions of interest (ROIs) (Fig. 1). ROIs for the femur included the femoral head, femoral neck, trochanter, shaft and distal femur. ROIs for the tibia included the proximal tibia, shaft and distal tibia. Nodes constrained at the distal end were excluded from the RMSE and NRMSE calculations for stress and strain.

Additionally, the linear correlations between shape/density errors and stress errors were analysed. Errors in bone shape were investigated between the SSDM-based and CT-based FE models using angular measurements (neck-shaft angle, anteversion angle and tibial torsion angle) (Supplementary Information S2). Density errors were quantified as node-to-node RMSE between SSDM-based and CT-based densities. Pearson correlation coefficients (*r*) were calculated to evaluate relationships between angular errors and stress errors, and between density errors and stress errors, with statistical significance set at *p* < 0.05.

Furthermore, the prediction accuracy of Von Mises stress and principal strains distributions were estimated using determination coefficients (R^2^) between the CT-based models and corresponding SSDM models. Each R^2^ value reflects the element-wise comparison of stress/strain pattern, providing a measure of how well the computed SSDM-based stress/strain distribution matches the CT-based stress/strain distribution at the element level. The stress distributions of models with highest and lowest R^2^ values were plotted for both the femur and tibia.

## Results

### Shape and density prediction accuracy

In the leave-one-out (LOO) analysis for the femur, the average RMS distance error between the predicted surface geometry from the SSDM-based model and CT-segmented surface mesh was 1.78 ± 0.46 mm. The average RMSE between the model predicted density and the CT-extracted density was 0.098 g/cm^3^, with an average NRMSE of 26.7%. More detail on the performance of the femoral statistical shape-density model can be found in (Xu et al. 2025). For the tibia, the average RMS distance error between the predicted model and CT-segmented surface mesh was 1.41 ± 0.39 mm. The average RMSE between the predicted density and CT-extracted density was 0.10931 g/cm^3^, with an average NRMSE of 28.7%. Further details about tibial SSDM prediction accuracy can be found in Supplementary Information S3 (Figs. S4, S5; Table S1).

**Fig. 4:**
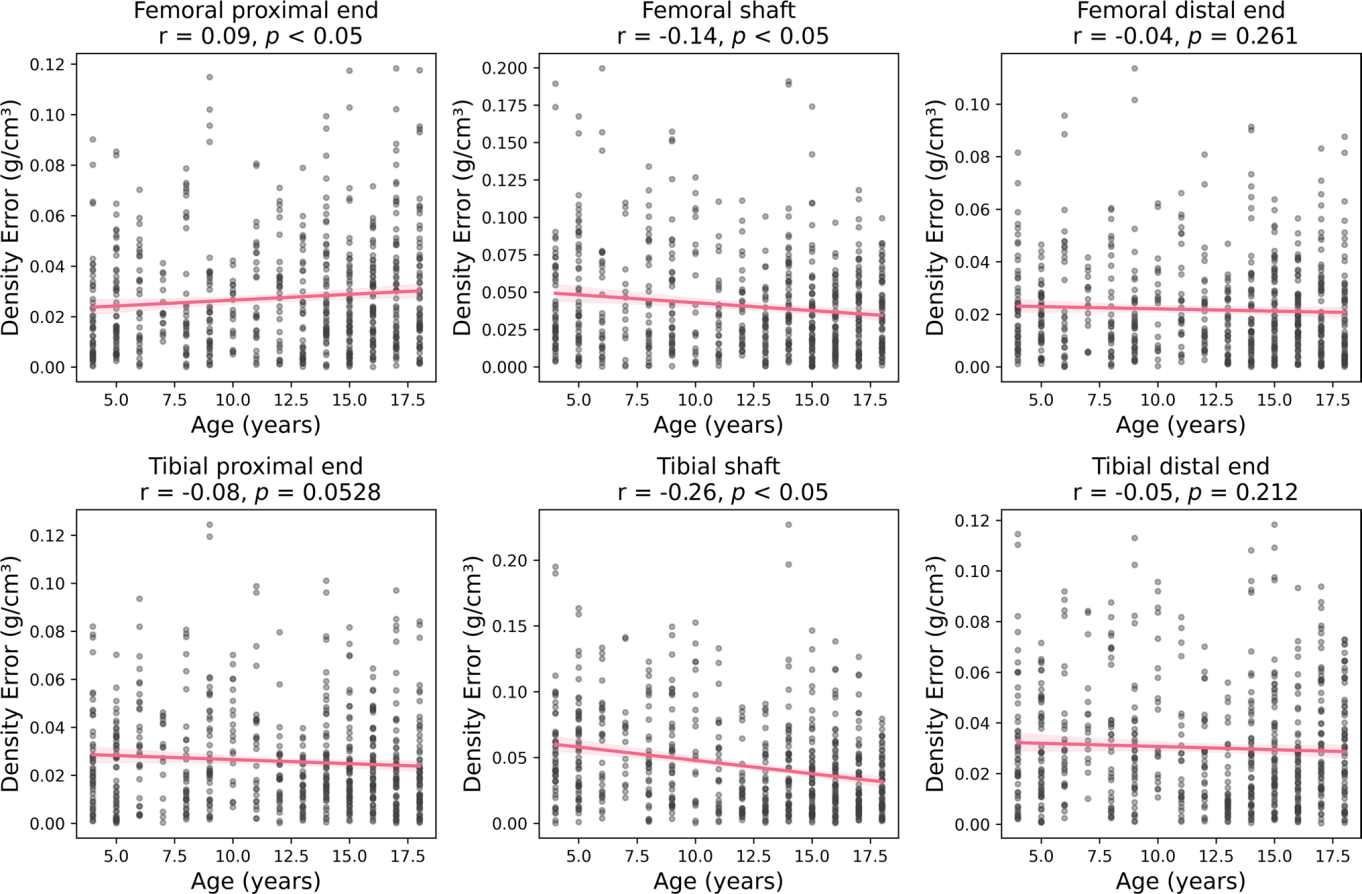
Correlation between age and absolute errors in bone mineral density across different regions, including proximal end, shaft and distal end in the femur and tibia. Pearson correlation coefficients (r) and *p*-values indicate the strength and statistical significance of correlations

**Fig. 5:**
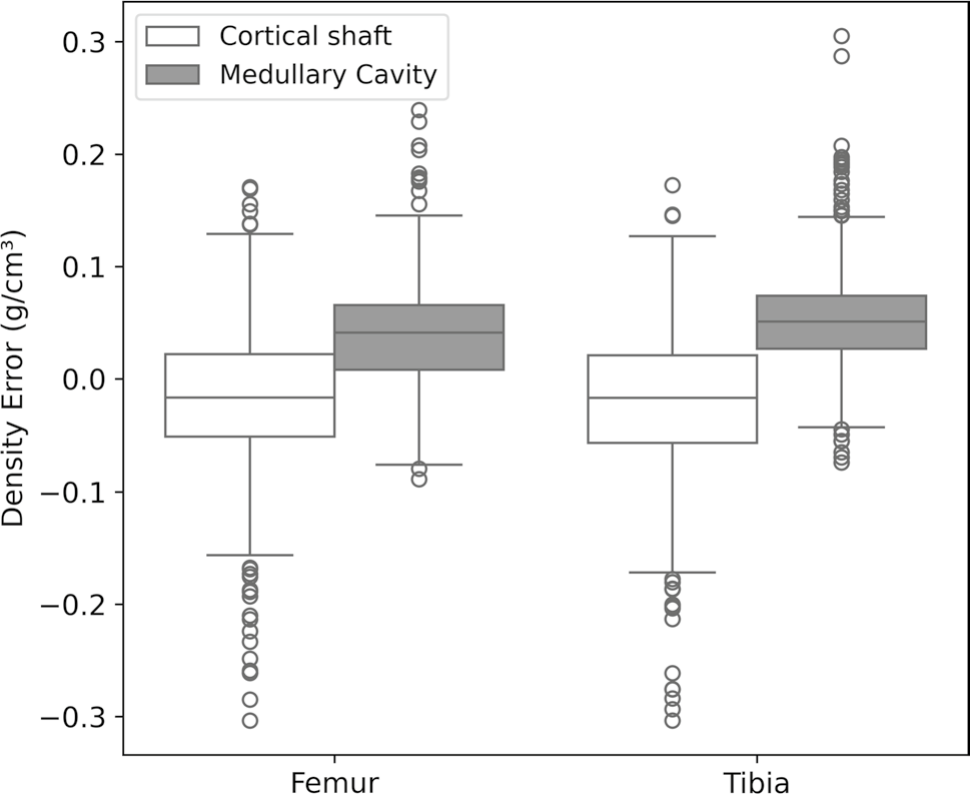
Boxplots showing density errors (predicted densities minus CT-based densities) in the cortical region and medullary cavity of the femoral and tibial shafts

**Fig. 6:**
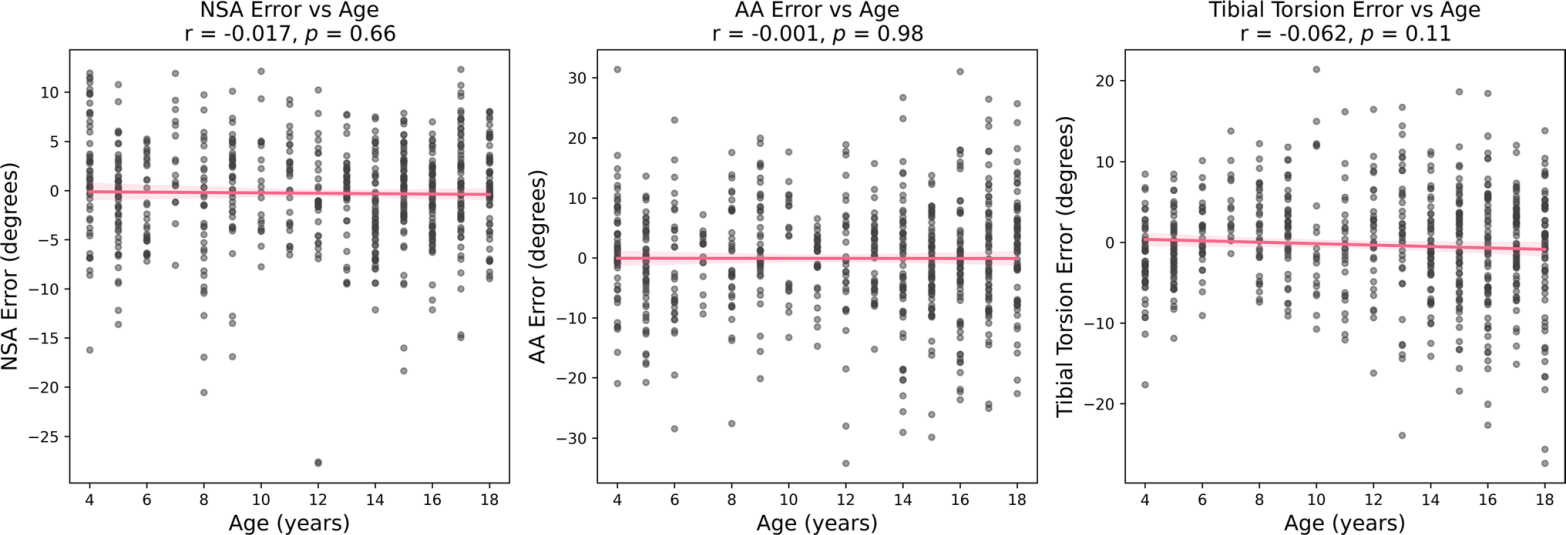
Correlations between bone angular measurement error and age. Pearson correlation coefficients (*r*) and *p*-values indicate the strength and statistical significance of the correlations

Correlations between regional density errors and age were found for the femur and tibia (Fig. 4). For the femur, a positive correlation was observed in the proximal end (*p* < 0.05), while a negative correlation was found in the shaft (*p* < 0.05). For the tibia, a negative correlation was noted in the shaft (*p* < 0.05), showing a systematic redistribution of density (Fig. 5), where cortical density is underestimated, and cavity density is overestimated. No correlation between bone angular measurement error and age was found (Fig. 6).

### Stress and strain prediction accuracy

The SSDM-based FEA closely approximated the CT-based results, with relative errors below 5% in average Von Mises stress and below 7% in average first principal strain for each ROI (Table 1). In contrast, maximum values exhibited larger discrepancies: relative errors in maximum stress ranged from − 16.3% in the proximal femur to 32.2% in the distal tibia, while maximum strain errors ranged from − 16.0% to 2.0%. A consistent underestimation of stress values was observed in the SSDM-based FEA compared to the CT-based FEA. Systematic bias and confidence intervals in Von Mises stress values were quantified using Bland–Altman plots and reported in Supplementary Information S4.

**Table 1:** Summary of average and maximum Von Mises stress and first principal strain (Mean ± Standard Deviation and ranges [Min–Max]) across femur and tibia regions, comparing CT-based and SSDM-based models

	Average Von Mises Stress (MPa)			Maximum Von Mises Stress (MPa)			Average first principal strain (µε)			Maximum first principal strain (µε)		
	CT-based FEA	SSDM-based FEA	Per cent error	CT-based FEA	SSDM-based FEA	Per cent error	CT-based FEA	SSDM-based FEA	Per cent error	CT-based FEA	SSDM-based FEA	Per cent error
Proximal Femur	1.94 ± 0.49 [0.96–4.15]	1.89 ± 0.44 [1.08–4.12]	− 1.4%	13.86 ± 2.97 [7.72–26.49]	11.52 ± 2.65 [6.82–26.61]	− 16.3%	354 ± 83 [192–719]	342 ± 64 [212–616]	− 1.8%	4646 ± 1246 [2290–10776]	4517 ± 715 [2908–7165]	1.5%
Femoral Shaft	2.57 ± 0.55 [1.44–4.90]	2.47 ± 0.49 [1.56–4.80]	− 3.1%	12.85 ± 2.78 [7.30–26.75]	10.04 ± 2.03 [6.11–19.60]	− 21.0%	117 ± 27 [74–285]	114 ± 21 [74–207]	− 0.6%	487 ± 138 [205–1177]	391 ± 73 [246–681]	− 16.0%
Distal Femur	0.92 ± 0.24 [0.49–2.13]	0.90 ± 0.20 [0.53–1.93]	− 0.7%	9.59 ± 3.05 [4.23–25.06]	7.46 ± 2.28 [3.56–18.72]	− 20.7%	110 ± 29 [59–263]	107 ± 19 [67–191]	− 0.3%	938 ± 374 [331–2786]	896 ± 268 [403–2194]	1.9%
Proximal Tibia	0.94 ± 0.22 [0.54–2.13]	0.92 ± 0.20 [0.58–1.99]	− 1.8%	10.78 ± 2.65 [5.44–26.06]	7.53 ± 1.72 [4.59–16.62]	− 29.0%	413 ± 136 [183–1339]	407 ± 80 [255–760]	4.2%	5223 ± 3135 [1315–22595]	4563 ± 1812 [1971–11154]	2.0%
Tibial Shaft	2.90 ± 0.68 [1.66–6.64]	2.77 ± 0.55 [1.77–5.21]	− 3.3%	14.46 ± 3.79 [8.40–43.90]	11.02 ± 2.39 [6.79–23.67]	− 22.0%	141 ± 36 [83–397]	147 ± 26 [96–251]	6.7%	437 ± 158 [212–1556]	417 ± 90 [246–820]	1.1%
Distal Tibia	1.66 ± 0.43 [0.88–3.72]	1.62 ± 0.38 [0.93–3.35]	− 1.6%	12.97 ± 3.71 [6.19–39.34]	8.65 ± 2.43 [4.51–20.62]	− 32.2%	337 ± 92 [177–858]	328 ± 54 [217–537]	1.6%	2486 ± 1320 [695–16416]	1950 ± 601 [987–4734]	− 11.5%

Relative errors are shown as percentages

**Fig. 7:**
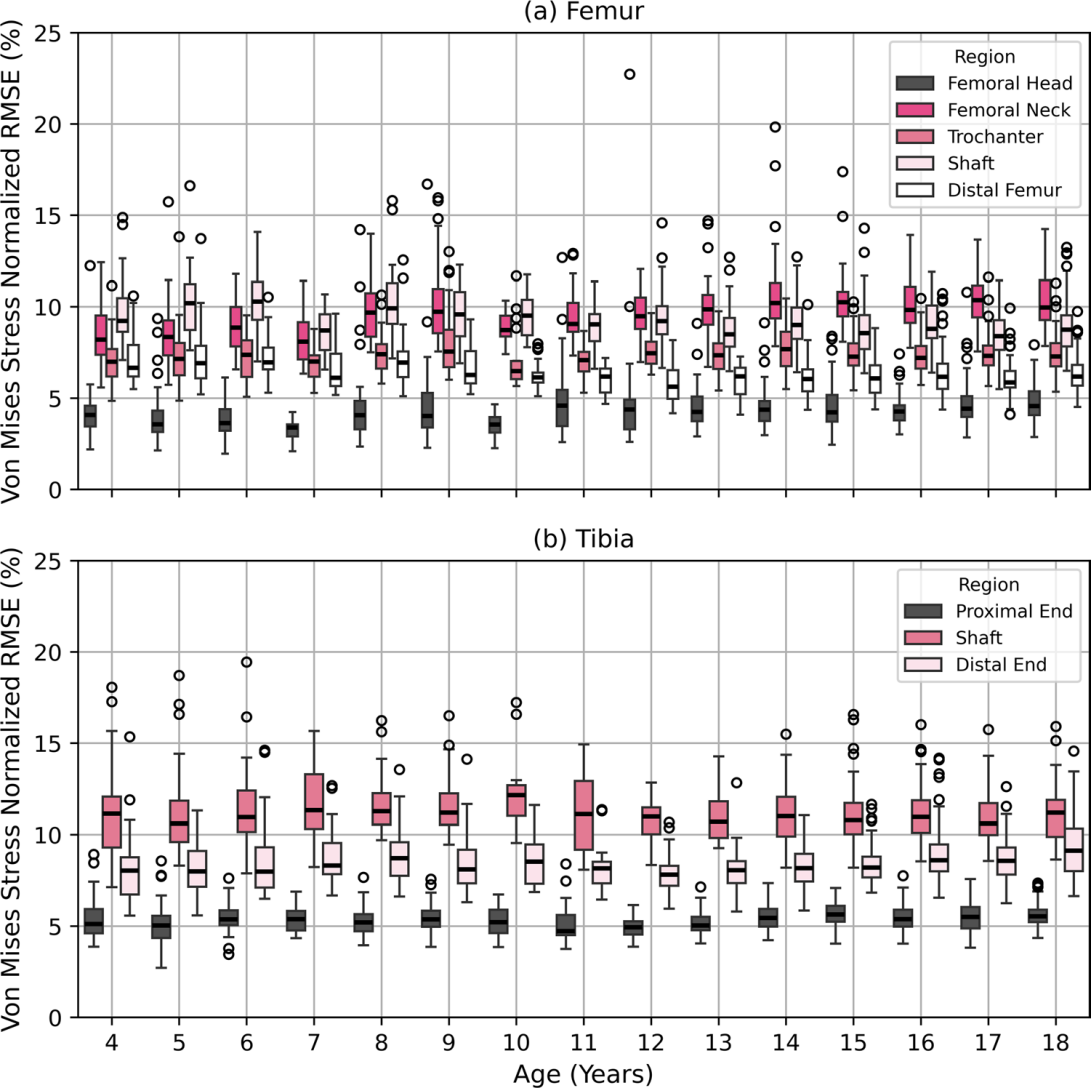
Von Mises stress root-mean-square error (RMSE) normalized by maximum stress in regions of interest in femur (**a**) and tibia (**b**) in the regions of interest (ROI). ROI in the femur included: femoral head, femoral neck, trochanteric region, shaft and distal femur; ROI in the tibia included: proximal end, shaft and distal end

The average Von Mises stress RMSE (NRMSE) between CT-based and SSDM-based FEA in the femur was 0.54 MPa (4.4%) for the femoral head, 0.89 MPa (9.8%) for the femoral neck, 0.98 MPa (7.4%) for the trochanteric region, 1.18 MPa (9.3%) for the shaft and 0.61 MPa (6.5%) for the distal femur. The NRMSE in the femoral neck exhibited a gradual increase with age, while a slight decreasing trend was observed in the shaft (Fig. 7a). Notably, the maximum NRMSE was observed in the femoral head region, reaching 22.7% (1.23 MPa).

For the tibial regions, the average Von Mises stress RMSE (NRMSE) were 0.58 MPa (5.4%) for the proximal end, 1.62 MPa (11.2%) for the shaft and 1.11 MPa (8.5%) for the distal end. The error stayed constant with age (Fig. 7b). The highest NRMSE of 19.4% was observed in the shaft region of a 6-year-old participant.

Overall, for the femur and tibia, the NRMSE in Von Mises stress were 6 and 8%, respectively. Furthermore, NRMSE in strains and maximum errors can be found in Supplementary information S5.

**Fig. 8:**
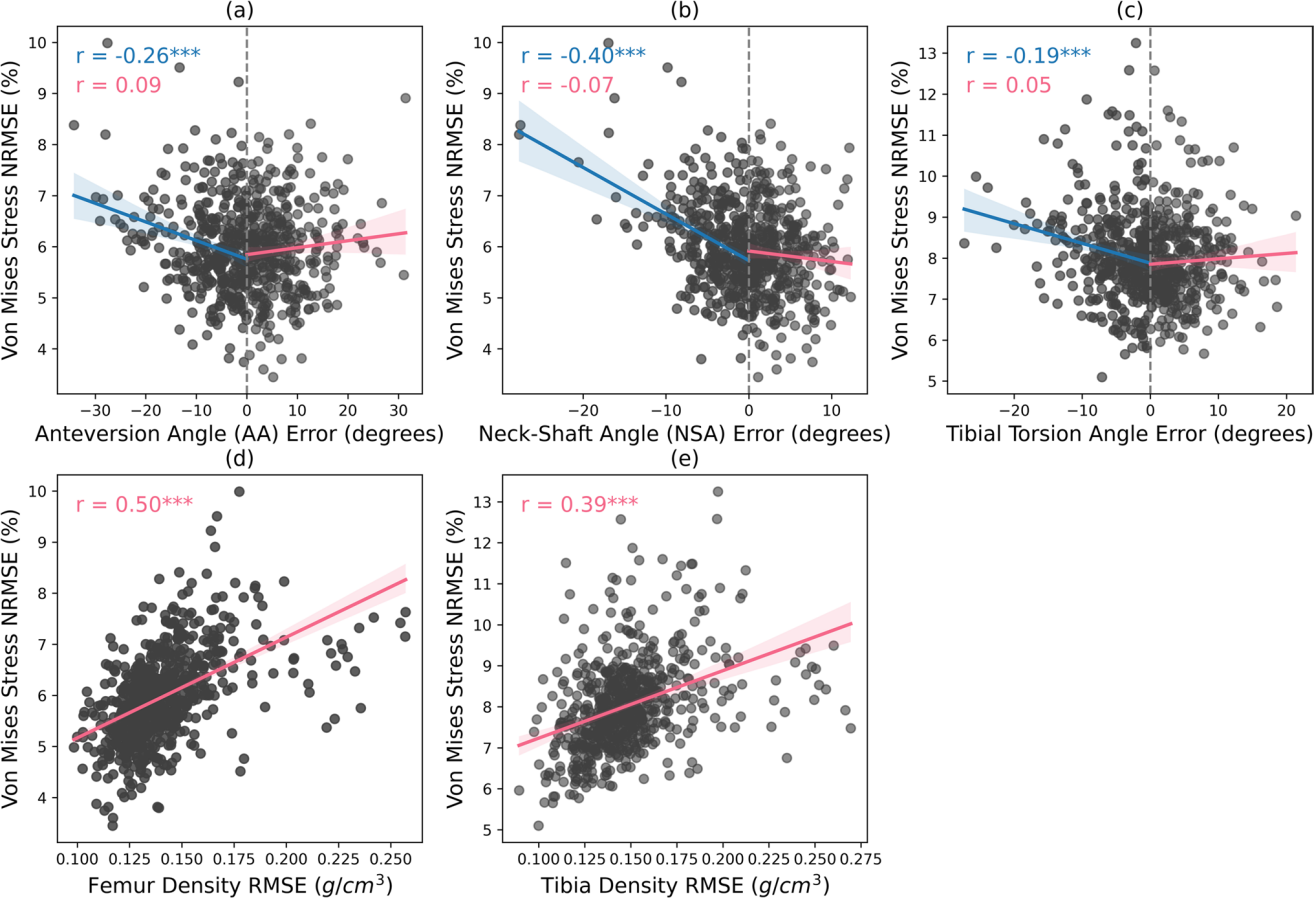
The correlations between Von Mises stress normalized root-mean-square error (NRMSE) and various femoral shape and density errors: **a** anteversion angle (AA) error; **b** neck-shaft angle (NSA) error; **c** tibial torsion angle error; **d** femoral density root-mean-square (RMSE) error between CT and shape-density model predictions and **e** tibial density RMSE between CT-based and predicted densities from shape-density model. Significant correlations are indicated by asterisks (*p < 0.05, **p < 0.01 and ***p < 0.001)

The underestimation of angular measurement errors was negatively correlated with Von Mises stress NRMSE, as shown in Fig. 8a–c. The strongest effect was observed for underestimated Neck Shaft Angle (NSA) error (*r* = − 0.40, *p* < 0.001). In contrast, density errors showed stronger positive correlations (Fig. 8d, e), with femoral stress NRMSE (*r* = 0.50, *p* < 0.001) and tibial stress NRMSE (*r* = 0.39, *p* < 0.001), indicating that greater density errors contributed to higher stress NRMSE.

**Fig. 9:**
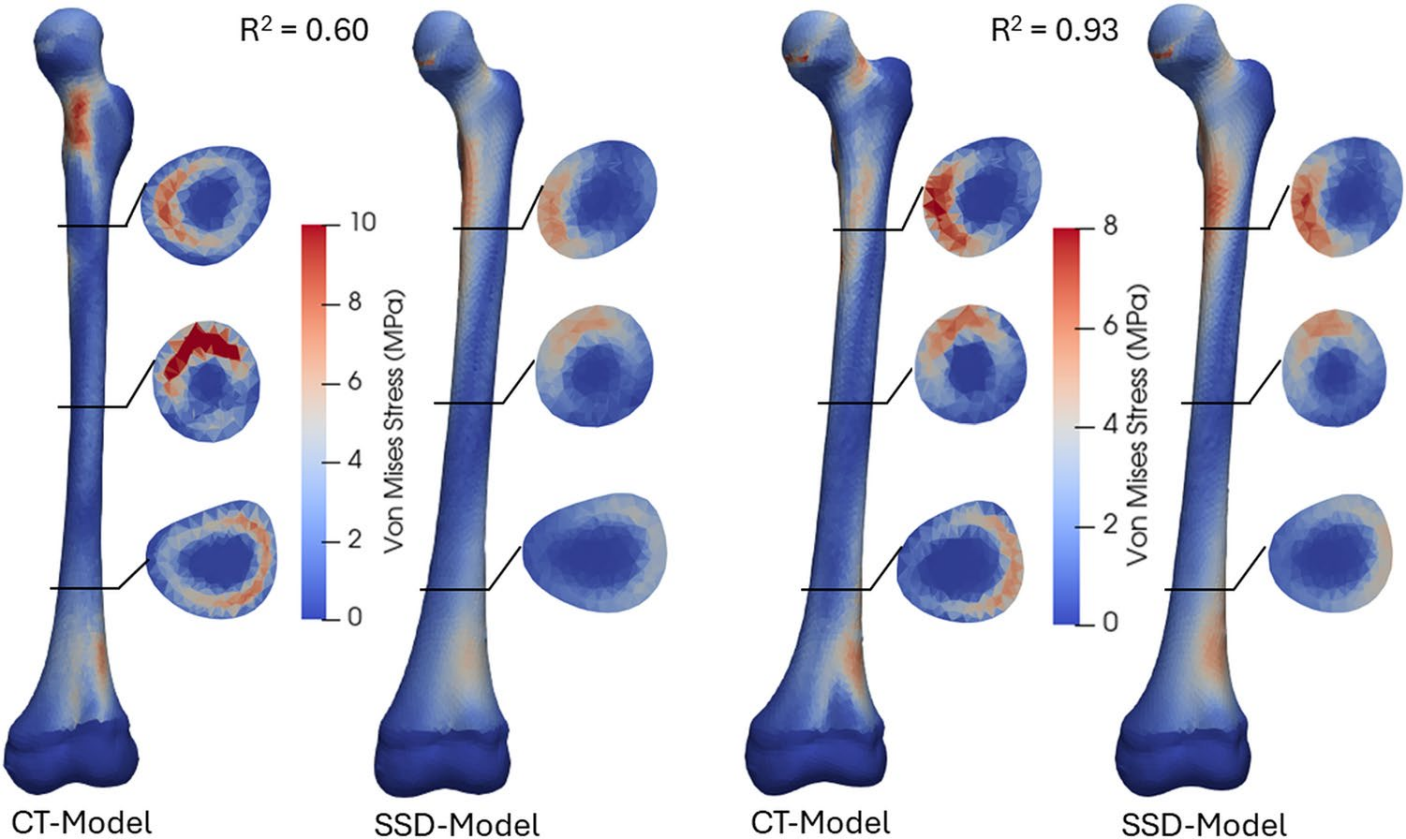
Von Mises stress distributions from CT-based FE and shape-density (SSD) FE models: anterior view of femur models with lowest (left) and highest (right) coefficients of correlation (*R*^2^). Cross-sections at 25, 50 and 75% of femoral total length

**Fig. 10:**
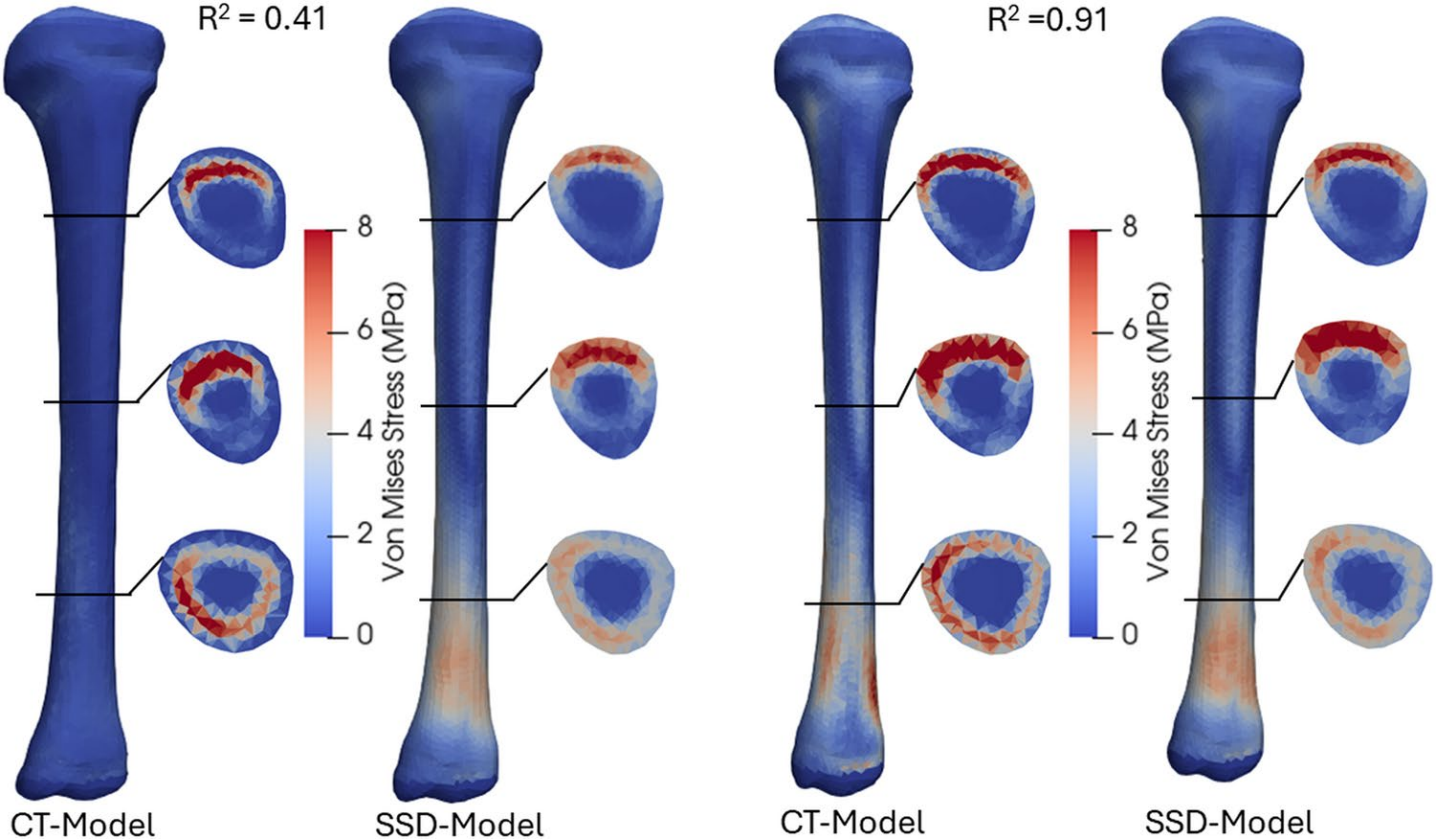
Von Mises stress distributions from CT-based FE and shape-density (SSD) FE models: anterior view of tibia models with lowest (left) and highest (right) coefficients of correlation (*R*^2^). Cross-sections at 25, 50 and 75% of tibial total length

High average determination coefficients (*R*^2^ ≥ 0.80) in stress and strain distributions were found between SSDM-based FEA and CT-based FEA in both the femur and tibia (Table 2). The Von Mises distributions and cross-sectional views of the models with the lowest and highest *R*^2^ values for the femur and tibia are shown in Figs. 9 and 10, respectively. The femur with the lowest *R*^2^ exhibited large shape errors (2.3 mm surface RMSE, − 16° neck-shaft angle, + 31° anteversion angle) and a large density error (0.148 g/cm^3^). In contrast, the tibia model with the lowest *R*^2^ had a minor shape error (1.48 mm surface RMSE, − 2° tibial torsion) but higher density error (0.196 g/cm^3^).

**Table 2:** The determination coefficient (*R*^2^) in stress and strain correlation analysis between CT-based FE models and SSDM-based FE models

	Femur		Tibia	
	Average *R*^2^	Range	Average *R*^2^	Range
Von Mises stress	0.84 ± 0.05	0.60–0.93	0.80 ± 0.07	0.41–0.91
1st principal strain	0.96 ± 0.02	0.70–0.99	0.86 ± 0.07	0.47–0.95
3rd principal strain	0.95 ± 0.02	0.77–0.99	0.85 ± 0.06	0.51–0.94

## Discussion

This study evaluated the potential of a statistical shape and density model (SSDM) to estimate stress and strain distribution compared to gold standard CT-based FEA in the paediatric femur and tibia.

The SSDM-based FE stress errors in this study were consistent with the previous studies on adult bones, with average stress errors ranging from 0.58 to 1.3 MPa (Taghizadeh et al. 2016) and a NRMSE between 10 and 18% (Nolte and Bull 2019). While the average stress values obtained from SSDM-based FEA closely matched those from CT-based FEA, maximum stress values were consistently underestimated across regions (Table 1). This trend aligns with the findings from Bruce et al. (2024), where SSDM-based FEA resulted in lower maximum strain estimates compared to CT-based models, especially in the tibial shaft. Bland–Altman analysis (Fig. S6) confirmed a systematic underestimation of mean stress differences in both femoral and tibial shafts. These findings are consistent with the study by (Taghizadeh et al. 2016) using machine learning models to predict femoral stresses during walking, where larger underestimated errors were found in the shaft region. Our results (Fig. 5) indicate that the observed underestimation of stress and strain values may be attributed to the underestimation of cortical density and overestimation of medullary cavity density, which likely result from uncertainties in defining the endosteal surface boundary in our model. Overall, our model demonstrated high accuracy with Von Mises stress NRMSE lower in the femur (6%) and the tibia (8%) compared to reported values for adult femurs (10–18%) (Nolte and Bull 2019).

Age-related variation in stress errors differed by region in the femur but remained stable in the tibia. In the femoral neck, Von Mises stress NRMSE increased with age, while the shaft showed a decreasing trend. These patterns were consistent with femoral density prediction errors which were positively correlated in the proximal region and negatively correlated in the shaft (*p* < 0.05; Fig. 4). Shaft density prediction accuracy improves with age, aligning with reduced standard deviation of CT-based density. Conversely, the increasing density variation in the proximal femur likely contributes to greater prediction error in older participants. These findings suggest that density prediction accuracy is influenced by the complex inter-individual deviations of the region-specific bone growth patterns in paediatric bones.

The maximum outlier in Von Mises NRMSE (22.7%) was found in the femoral head region. This outlier corresponded to a femoral model based on a 12-year-old participant, who exhibited a relatively high neck-shaft angle (≥ 160°) and a large anteversion angle (≥ 40°), when compared to the angles reported in the previous study (Carman et al. 2023). The shape-density model underestimated the neck-shaft angle by 27.7° and underestimated the anteversion angle by 28° for this participant. These shape errors influenced the Von Mises stress distributions, showing negative correlations with stress errors (Fig. 4a, b). In addition to the shape errors, this model also had a large density prediction error of 0.148 g/cm^3^, which likely contributed to the high stress NRMSE observed. Prior studies (Oh et al. 2017; Kim and Kim 2021) support these findings that the neck-shaft angle influences both the magnitude and location of peak stresses. However, contrary to the study by (Politis et al. 2013), we observed that overestimated anteversion deviations had a weak influence on stress errors, whereas their study suggested that deviations in either direction elevate and redistribute stresses. While underestimated angles in femoral shape moderately influenced stress distributions, shape errors overall had a weaker impact on stress errors compared to density errors, which showed stronger correlations with stress prediction accuracy (Fig. 8). Our findings highlight the limitations of using demographics and bone linear measurements in the selected prediction method (Partial Least Square Regression) in accurately predicting extreme neck-shaft and anteversion angles, contributing to higher stress errors. Incorporating further clinical information such as partial medical imaging would improve prediction performance, particularly for individuals with extreme femoral measurements.

The maximum stress NRMSE recorded in the tibia was observed in the shaft (19.4%) of a model with an underestimated tibial torsion of 2° and a density error of 0.195 g/cm^3^ (68%). Our findings highlight the dominant influence of density error on the stress prediction accuracy in the tibia rather than angular measurements (Fig. 8).

CT-based FEA, widely recognized as the gold standard, has demonstrated excellent accuracy, with R^2^ values for principal strains over 0.94 when validated against experimental data (Grassi et al. 2016). In our study, high coefficient of determination (*R*^2^) was achieved for both the femur and tibia when comparing results of SSDM-based FEA to those of the CT-based gold standard. On average, femoral *R*^2^ values were over 0.80 for stress and 0.85 for strain. While tibial predictions were slightly less accurate than femoral predictions, they remained within a high accuracy range. Additionally, our model outperformed generic FEA approaches, which have reported strain *R*^2^ values ranging from 0.48 to 0.92 across femoral regions when compared to CT-based FEA (Martelli et al. 2015). Given the high level of correlation achieved (*R*^2^ ≥ 0.8) and low NRMSE (< 10%), our results suggest that our SSDM-based FE models offer a practical alternative for performing FEA in paediatric population. This level of agreement is relevant for applications such as pre-surgical planning, where the primary goal is often to understand relative stress/strain distributions, or compare the biomechanical effects of different surgical strategies, especially when CT-based models are unavailable or undesirable due to radiation exposure.

Several limitations of the current study should be mentioned. First, contact forces were derived from average values reported in in vivo studies from the adult OrthoLoad database, and muscle forces were not incorporated into the simulation. While we anticipate that the relative differences observed between the SSDM-based and CT-based FE models would remain consistent, inclusion of more personalized boundary conditions would influence the absolute values of stress and strain. Our current study focused on the stress/strain distributions comparison between SSDM-based and CT-based FEA, so we explored a single boundary condition (single-leg standing). Future work will assess the influence of different loading scenarios on stress and strain distributions in SSDM- and CT-based models to enhance the physiological relevance of the comparison. Second, the relationships between the ash density and apparent density, as well as between CT density and Young’s modulus, were derived from studies on adults which might not be a good representation for our paediatric population. Further research is needed to validate these relationships in a paediatric population, as this will influence the stress–strain magnitudes. Additionally, we were unable to isolate the effects of prediction errors in geometry and material properties on stress and strain accuracy, as these factors are interconnected in the current shape-density model. Future studies should aim to control these variables independently to understand their individual influence on stress and strain distribution.

In conclusion, this study demonstrated that paediatric SSDM-based FE models generated from shape and density model can reproduce stress and strain distributions compared to CT-based FEA (gold standard) with strong correlations and errors within 8% in the femur and tibia. Our findings support the potential applicability of SSDM-based FE models to serve as surrogate for CT-based FE models in clinical settings, particularly where CT imaging is limited due to radiation concerns in paediatric populations. To promote open science and facilitate future research in paediatrics, the SSDMs in current study will be openly accessible on SimTK (https://simtk.org/projects/paed_ssm). Future research will focus on improving prediction accuracy by integrating additional reference points or partial medical imaging, which might be available as part of pre-surgery clinical care. This advancement could establish SSDM-based FE models as valuable tools for personalized implant design and surgical planning for paediatric populations.

## Supplementary Information

Below is the link to the electronic supplementary material.

Supplementary file1 (DOCX 769 KB)

## Data Availability

No datasets were generated or analysed during the current study.
