# Supplementary material for: Prediction accuracy of femoral and tibial stress and strain using statistical shape and density model-based finite element models in paediatrics
Source: Biomech Model Mechanobiol. 2025 Oct 13;24(6):2237–49. doi: 10.1007/s10237-025-02016-8 (PMC12618425; doi:10.1007/s10237-025-02016-8)
Supplement: Supplementary file 1 — Supplementary file1 (DOCX 769 KB) [file 10237_2025_2016_MOESM1_ESM.docx]

Evaluation of Personalized Finite Element Models Based on Statistical Shape and Density Models for Predicting Femoral and Tibial Stress and Strain in Paediatrics

**Authors:** Yidan Xu^a^; Laura Carman^a^; Thor Besier^a,b^; Julie Choisne^a*^

a. Auckland Bioengineering Institute, The University of Auckland, Auckland, New Zealand.

b. Department of Engineering Science and biomedical engineering, The University of Auckland, Auckland, New Zealand.

***Corresponding Author:**

Julie Choisne, PhD

70 Symonds Street, Auckland, NZ

j.choisne@auckland.ac.nz

S1. Tibia mesh morphing quality

Mesh quality of the morphed meshes was assessed using average and standard deviations of the radius ratio (Fig. S1). The average quality values of morphed meshes was 1.50 ± 0.05. These values were comparable to those of the template meshes 1.38 ± 0.49, indicating that the quality of the morphed meshes was maintained. Some morphed meshes exhibited higher radius ratio values than the template. Specifically, the maximum radius ratio values for the morphed meshes was 1.71 ± 1.3, which is from the left femur of a 15-year-old participant.

(a)


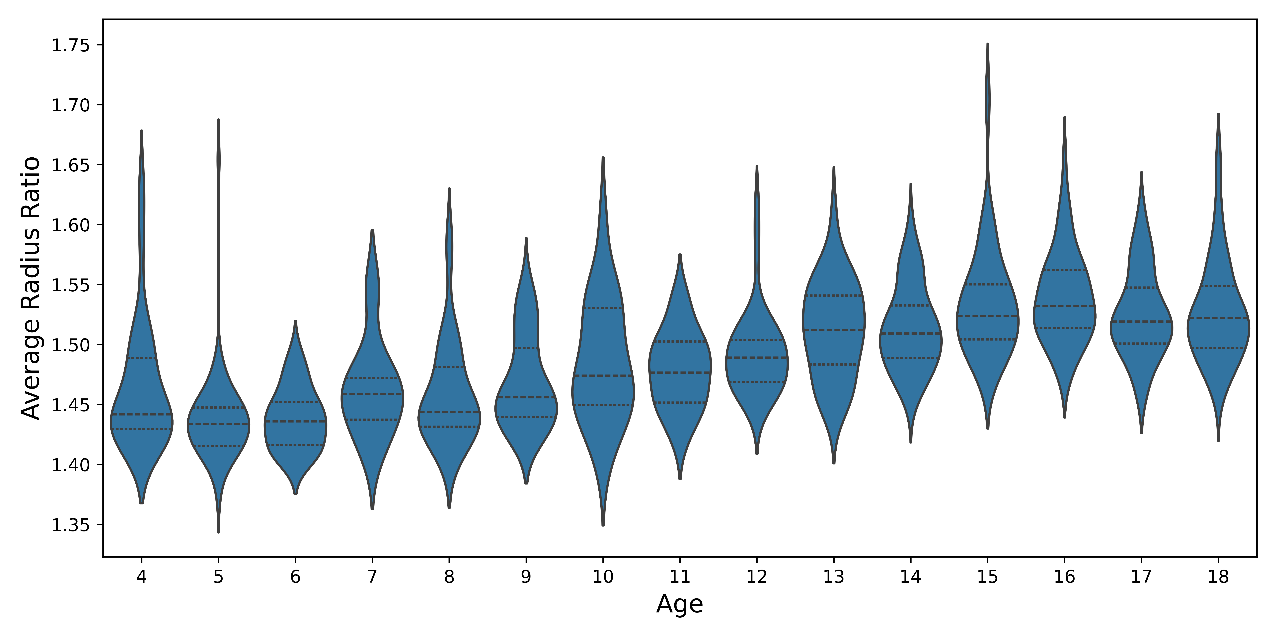


(b)


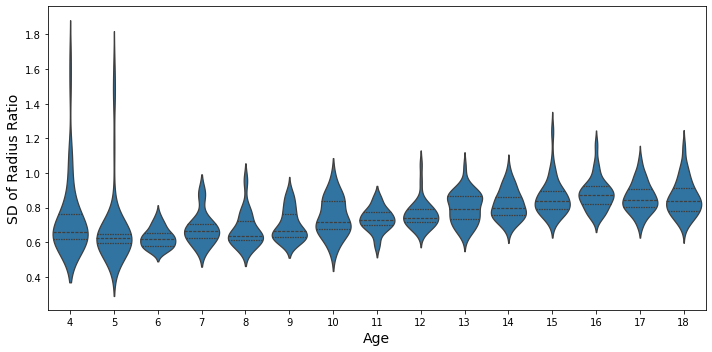


**Fig. S1** Average values (a) and standard deviation (b) of radius ratio in morphed meshes for the tibia distributed by age

S2. Femoral and tibial morphology measurements

Linear bone measurements were defined as shown in Fig. S2.

Computed linear measurements included femoral length, epicondylar width, condylar width, tibial length, and malleolar width (Fig. S2). Linear bone measurements were taken from automatically determined bony landmarks for each bone as described in (Carman et al. 2023). The femoral length was determined by the distance between the greater trochanter and the lateral epicondyle bony landmarks. The tibial length was determined by the distance between the lateral condyle and the lateral malleolus of the tibia.


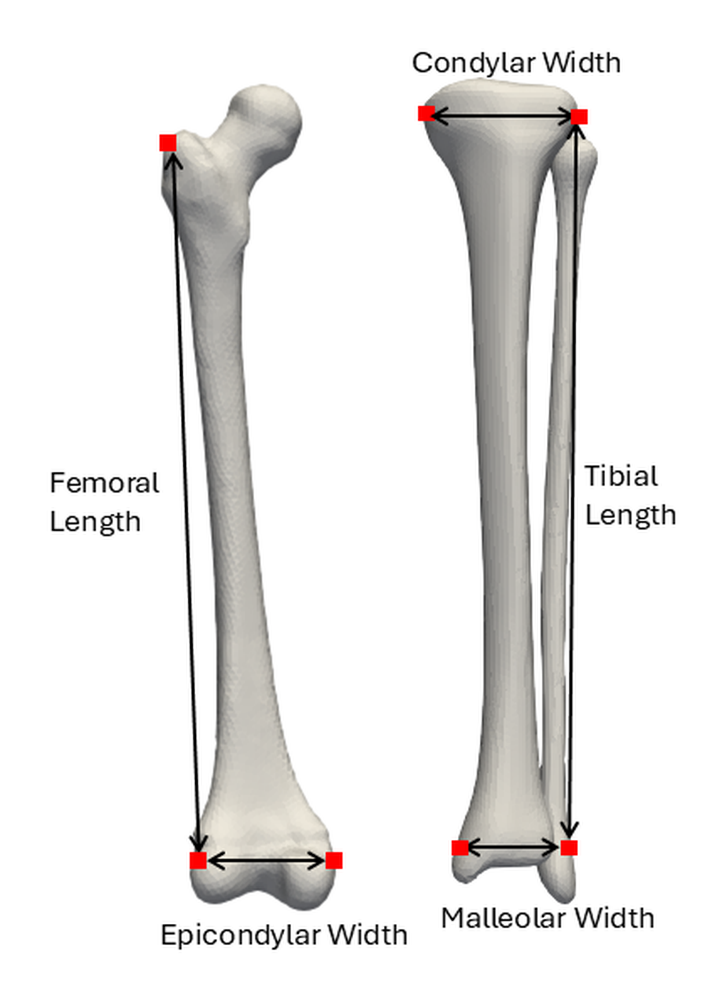


**Fig. S2** Linear measurements in the femur and tibia as described in (Carman et al. 2023)

Angular and torsional measurements were defined as shown in Fig. S3.

Neck-shaft angle (Carman et al. 2023): angle between the neck axis of the femur and the shaft axis of the femur (measured between the centre of a cylinder fit to the femoral shaft below the lower trochanter and the midpoint of the centre of a cylinder fit to each femoral condyle).

Anteversion angle (Carman et al. 2023): angle between the neck axis of the femur (measured between a sphere fit to the femoral head and the centre of a cylinder fitted to the femoral neck), and the posterior condylar axis (measured between the medial and lateral posterior femoral condyle).

Tibial Torsion (Chen 2020): angle between the posterior condylar axis (measured between the medial and lateral posterior tibial condyle) and distal reference axis (measured between the center point of the distal tibia and the midpoint of a line across the fibular notch of the tibia).


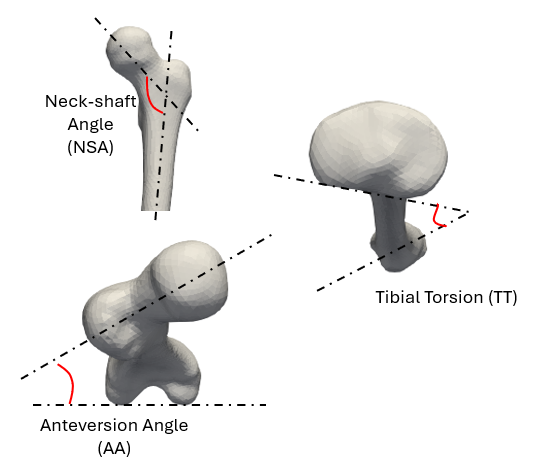


**Fig. S3** Angular and torsional measurements in the femur and tibia

S3. Assessment of the statistical shape and density model prediction accuracy in the tibia

The first 27 PCs captured over 90% of the shape and density variance in the tibia. Beyond 27 PCs, the variance explained by each additional PC dropped to just 0.22%. At 27 PCs, the compactness analysis yielded an average RMSE of 1.08 mm for the shape, and 0.0675 g/cm^3^ for the density, indicating sufficient variance representation (Fig. S4). Furthermore, the specificity study confirmed that the prediction accuracy did not improve significantly beyond 27 PCs, and no overfitting was observed within this range (Fig. S5). In addition, the estimated Young’s Modulus, corresponding to the density predicted with 27 PCs, remained below 172 MPa, based on equations (4)-(6) in this paper. Consequently, we selected 27 PCs for further leave-one-out analysis to achieve a balance between accuracy and overfitting.


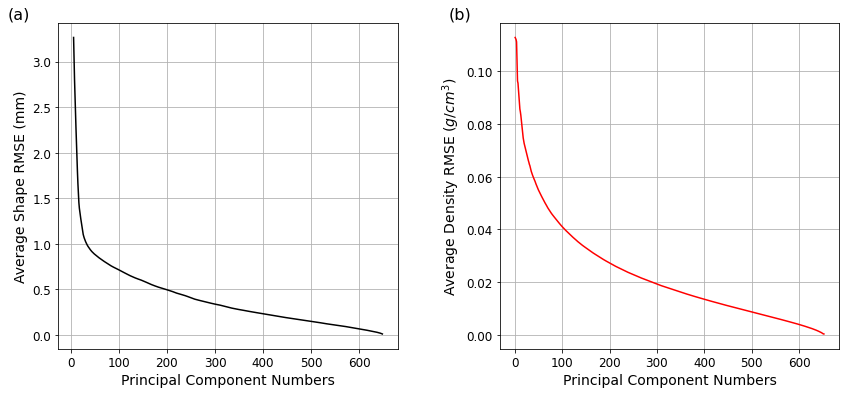


**Fig. S4** Compactness analyses showing the tibial shape prediction RMSE relative to the number of PC included (a) and the density prediction RMSE (b) relative to the number of PC included


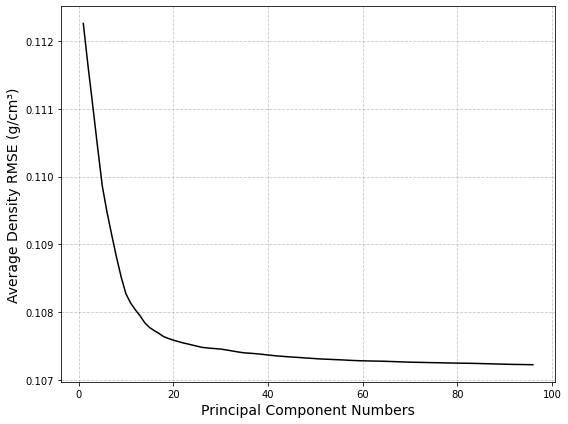


**Fig. S5** Specificity study results showing density RMSE relative to the number of PC included

In the leave-one-out (LOO) analysis, the average RMS distance error between the predicted and segmented surface mesh was 1.41 ± 0.39 mm. In addition, the average RMSE for density across nodes was 0.10931 g/cm^3^, with a normalized RMSE of 28.7%. We also investigated the difference (original density - predicted density) in the density per region in the tibia and found errors were lower for each region with the shaft having the lowest normalized prediction error (Table S1).

**Table S1** Bone mineral density prediction error (mean error in g/cm^3^ and normalized error (%)) for the leave-one-out analysis. The errors are displayed for the node-to-node comparison and average error in each region of interest

| Regions of Interest | Mean Error (g/cm^3^) | Normalized Error (%) |
| --- | --- | --- |
| Node-to-node comparison | 0.109 | 28.7% |
| Proximal end | 0.026 | 11.5% |
| Shaft | 0.044 | 7.9% |
| Distal end | 0.030 | 10.5% |

S4. Systematic bias in Von Mises stress

Systematic bias and confidence intervals in Von Mises stress values were quantified using Bland-Altman plots in specific regions: proximal end (upper 20% of total length), shaft and distal end (lower 20% of total length) for both femur and tibia (Gosman et al. 2013).

The Bland-Altman plots (Fig. S6) illustrated the agreement between CT-based and SSDM-based FEA in predicting Average Von Mises stress across different regions of the femur and tibia, including proximal end, shaft and distal end. Systematic bias were found at the shaft, where the mean differences were negative, indicating that the SSDM-based FEA underestimated the Von Mises stress compared to CT-based FEA. Smaller bias and discrepancies were observed at the proximal and distal ends of both bones. Overall, there was good agreement between the models, with observed differences in Von Mises stress up to -0.13 MPa and discrepancies up to 0.76 MPa across the various regions.


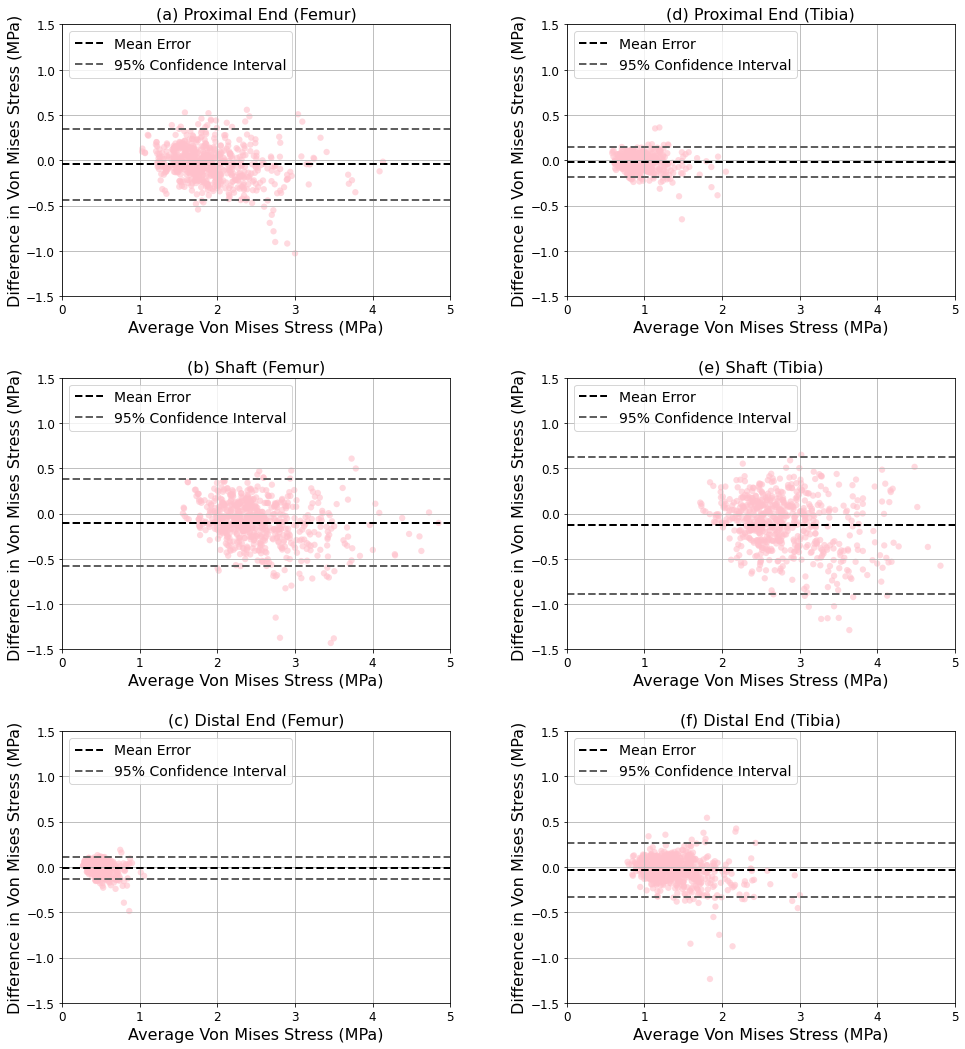
**Fig. S6** Bland-Altman plots of Von Mises stress for the femur and tibia: average Von Mises stress of CT-based and SSDM-based models against their differences across different regions: proximal femur (a), femoral shaft (b), distal femur (c), proximal tibia (d), tibial shaft (e), distal tibia (f)

S5. Stress and strain prediction accuracy

Von Mises stress differences were assessed at the element level using root-mean-square error (RMSE) and normalized root-mean-square error (NRMSE), which was normalized by the maximum stress. In addition to stress, RMSE and NRMSE were assessed for principal strains across the femur and tibia. Maximum absolute error was also calculated to evaluate stress and strain differences. Maximum absolute error (percent) was normalized by the maximum value.

**Table S2** Average root-mean-square error (RMSE), normalized RMSE (NRMSE), maximum error and normalized maximum error between CT-based and SSDM-based models in femur

|  | RMSE | NRMSE | Max Absolute Error | Max Absolute Error (percent) |
| --- | --- | --- | --- | --- |
| Von Mises stress | 0.85 MPa | 6.0 % | 6.85 MPa | 48.9 % |
| 1^st^ principal strain | 54 µε | 1.2 % | 1217 µε | 26.3 % |
| 3^rd^ principal strain | 86 µε | 2.3 % | 1288 µε | 33.1 % |

**Table S3** Average root-mean-square error (RMSE), normalized RMSE (NRMSE), maximum error and normalized maximum error between CT-based and SSDM-based models in tibia

|  | RMSE | NRMSE | Max Absolute Error | Max Absolute Error (percent) |
| --- | --- | --- | --- | --- |
| Von Mises stress | 1.18 MPa | 8.0 % | 8.23 MPa | 55.5 % |
| 1^st^ principal strain | 109 µε | 2.4 % | 2551 µε | 50.9 % |
| 3^rd^ principal strain | 225 µε | 5.5 % | 2279 µε | 52.5 % |

Reference

Carman L, Besier T, Stott NS, Choisne J (2023) Sex differences in linear bone measurements occur following puberty but do not influence femoral or tibial torsion. Sci Rep 13:11733. https://doi.org/10.1038/s41598-023-38783-6

Chen BPJ (2020) Measuring Femoral and Tibial Torsion in Children with Cerebral Palsy. In: Cerebral Palsy: Second Edition. Springer International Publishing, pp 1381–1400

Gosman JH, Hubbell ZR, Shaw CN, Ryan TM (2013) Development of Cortical Bone Geometry in the Human Femoral and Tibial Diaphysis. Anatomical Record 296:774–787. https://doi.org/10.1002/ar.22688
